# Integrating alpha, beta, and phylogenetic diversity to understand anuran fauna along environmental gradients of tropical forests in western Ecuador

**DOI:** 10.1002/ece3.5593

**Published:** 2019-09-12

**Authors:** Luis Amador, Mauricio Soto‐Gamboa, Juan M. Guayasamin

**Affiliations:** ^1^ Doctorado en Ciencias mención Ecología y Evolución Facultad de Ciencias Universidad Austral de Chile Valdivia Chile; ^2^ Universidad Laica Vicente Rocafuerte de Guayaquil Guayaquil Ecuador; ^3^ Instituto de Ciencias Ambientales y Evolutivas Facultad de Ciencias Universidad Austral de Chile Valdivia Chile; ^4^ Instituto de Investigaciones Biológicas y Ambientales BIÓSFERA Laboratorio de Biología Evolutiva Colegio de Ciencias Biológicas y Ambientales COCIBA Universidad San Francisco de Quito USFQ Quito Ecuador; ^5^ Centro de Investigación de la Biodiversidad y Cambio Climático Ingeniería en Biodiversidad y Recursos Genéticos Facultad de Ciencias de Medio Ambiente Universidad Tecnológica Indoamérica Quito Ecuador

**Keywords:** amphibia, Andes, phylogenetic structure, species richness, turnover, variation

## Abstract

The study of current distribution patterns of amphibian species in South America is of particular interest in areas such as evolutionary ecology and conservation biology. These patterns could be playing an important role in biological interactions, population size, and connectivity, and potential extinction risk in amphibians. Here, we tested the effects of spatial and environmental factors on the variation, turnover, and phylogenetic diversity of anuran amphibian species in tropical forests of western Ecuador. Data for presence/absence of 101 species of 34 genera and 10 families registered in 12 sites (nested in four biogeographic units) were obtained through fieldwork, museum collections, and literature records. We examined the influence of geographical, altitudinal, temperature, and precipitation distances on differences in anuran composition between sites. We found significant positive correlations among all of these variables with anuran distribution. The greatest alpha diversity (species richness) was found in the Equatorial Chocó biogeographic unit. Equatorial Pacific biogeographic unit could act as a transition zone between the Equatorial Chocó and Equatorial Tumbes. The western Andes (Western Cordillera biogeographic unit) was the most dissimilar and exhibited a higher species turnover rate than the other biogeographic units. Our results suggest that precipitation and elevation play a key role in maintaining the diversity of amphibian species in western Ecuador.

## Graphical abstract

Environmental factors affecting actual distribution can help to understanding biological interactions, future direction of populations, and potential extinction risk of species. In this work, we studied the effect of geographical and environmental factors on species turnover and phylogenetic structure of amphibian anurans in tropical forests of western Ecuador. Changes in amphibian diversity were analyzed by considering the phylogenetic distance between species of anurans. We examine the influence of altitude, temperature, and precipitation between sites on distribution patterns of anurans, showing that there is a significant positive correlation in all these variables. Our results suggest that species turnover, phylogenetic diversity can be explained by the high environmental heterogeneity present in the study area.

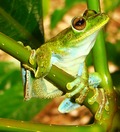

## 1 INTRODUCTION

Understanding the influence of environmental factors on community membership is an essential part of determining how species are distributed in space (Gaston, 2000; Stein, Gerstner, & Kreft, 2014). Explaining the distribution patterns of species in terms of environmental variables can provide insights on the operational limits of species in their distributional areas (Gotelli et al., 2009; Wiens, 2011). Communities are not just random groups of species; therefore, in a biogeographic area, there are may be a variable number of communities, which are composed of species that share or compete for habitat resources. (Cornell & Lawton, 1992; Wiens, 2011). Thus, community patterns are better explained by integrating both environmental and ecological variables when determining biogeographic patterns at different scales (Jiménez‐Robles, Guayasamin, Ron, & De la Riva, 2017; Wiens, 2011).

Amphibians have, on average, smaller body sizes than other terrestrial vertebrates, thus enabling them to occupy relatively narrow niches unavailable for larger vertebrates (Wells, 2007). This in turn could cause that assemblages of amphibians are the most vulnerable and less tolerant to environmental changes (Blaustein et al., 2010; Duarte et al., 2012; Navas & Otani, 2007). Taking into account the accelerated transformation of natural ecosystems (Geist & Lambin, 2002; Lambin & Meyfroidt, 2011), an understanding of how the diversity of amphibians is distributed and composed is essential for amphibian diversity conservation. Amphibians, mainly anurans species, face serious threats due to the combined effects of climate change, habitat loss, and diseases spread (Almeida‐Gomes, Vieira, Duarte Rocha, Metzger, & Coster, 2016; Berger et al., 1998; Blaustein & Bancroft, 2007; Jongsma, Hedley, Duräes, & Karubian, 2014; Lessmann, Muñoz, & Bonaccorso, 2014; Lips et al., 2006; Pounds et al., 2006; Stuart et al., 2004).

Studies from a global perspective have been carried out to analyze how the richness and turnover of amphibian species respond to different environmental and spatial gradients (Buckley & Jetz, 2007, 2008) and also to test the influence of phylogenetic history on the global patterns of amphibian species richness (Fritz & Rahbek, 2012). Recent studies incorporating geographic, ecological, and biological variables as well as phylogenetics have been conducted to resolve the biogeography of amphibians globally and within the Neotropical region (e.g., Castroviejo‐Fisher, Guayasamín, González‐Voyer, & Vila, 2014; Gonzalez‐Voyer, Padial, Castroviejo‐Fisher, De la Riva, & Vilá, 2011; Hutter, Lambert, & Wiens, 2017; Jiménez‐Robles et al., 2017; Pinto‐Sánchez, Crawford, & Wiens, 2014). Despite this, research on Neotropical biota often emphasizes the influence of historical factors rather than ecological factors when determining species distributions (Wiens, 2011). Ecological factors are key when describing assemblage patterns especially in areas with high species richness and turnover rates (e.g., Ecuador with 600 amphibian species, see Centro Jambatu, 2011–2017). Furthermore, the diversity of habitats occupied by amphibian assemblages has influenced the phylogenetic diversity of this group in the Neotropics (Arteaga et al., 2016; Jiménez‐Robles et al., 2017; Ribeiro, Colli, Batista, & Soares, 2017). Overall, studies of the spatial patterns of species assemblages are urgently required to delineate conservation strategies in ecosystems under strong anthropogenic pressures such as the evergreen forests of Chocó and Equatorial dry forests, two of the most fragmented forests in western Ecuador (Dodson & Gentry, 1991; Escribano‐Avila et al., 2017).

Here, we analyzed the community composition and phylogenetic structure of anurans occurring in 12 sites of four biogeographic units of western Ecuador. For this purpose, we use the alpha, beta, and phylogenetic diversity in order to establish questions about the factors that determine the variation of the diversity of anurans between biogeographic units of western Ecuador. We hypothesized that diversity (alpha, beta, and phylogenetic diversity) would depend on local‐level composition of species in the sites and their location along environmental gradients. In summary, this study provides a baseline of the phylogenetic diversity of anuran species in western Ecuador, allowing us to propose “hot spots” of amphibian diversity in this region.

## 2 MATERIALS AND METHODS

### 2.1 Study area and biogeographic units

**Figure 1:**
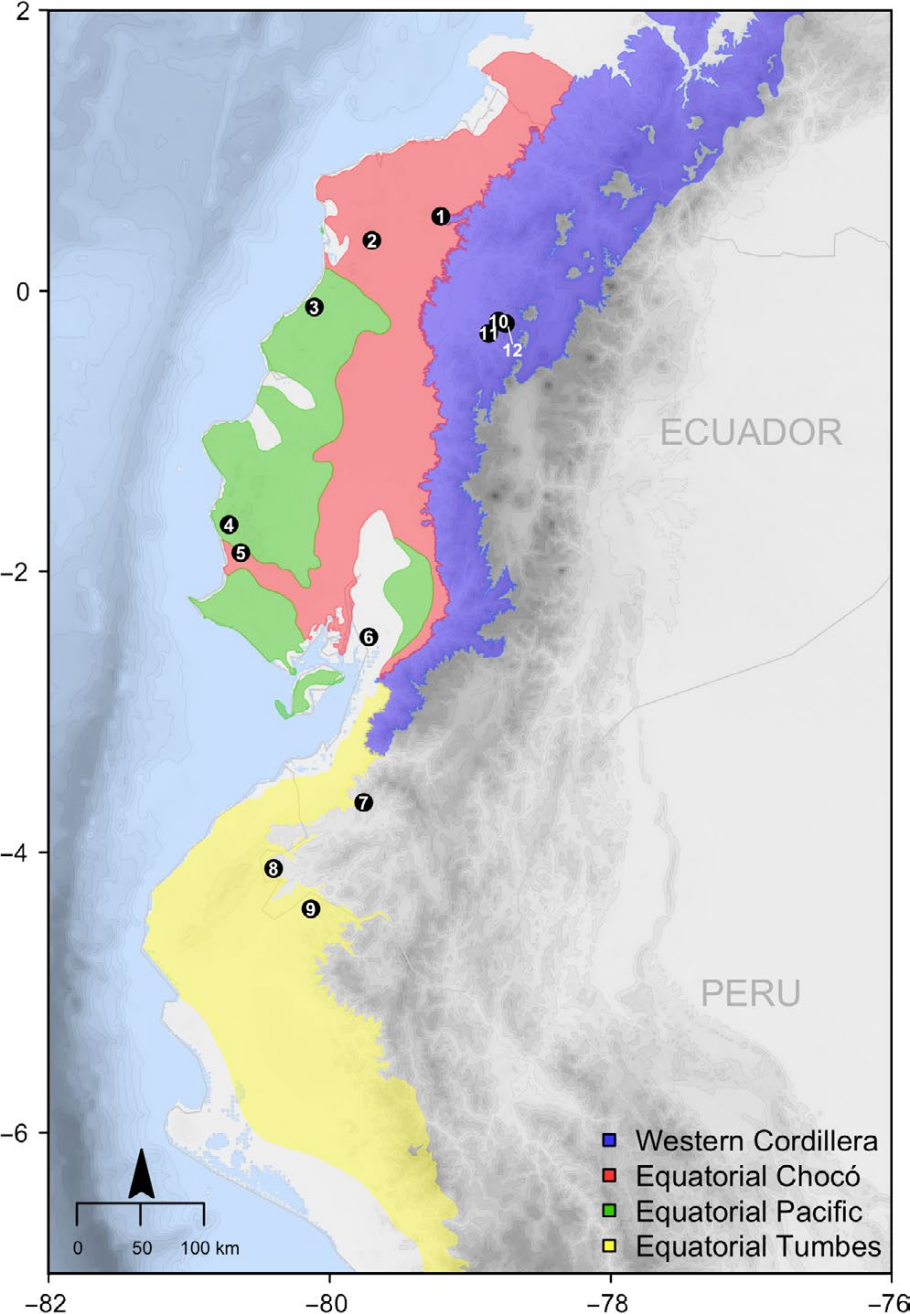
Map of Ecuador showing the study sites. 1 = Río Canandé, 2 = Bilsa‐Mache Chindul, 3 = Jama‐Coaque, 4 = Ayampe‐Machalilla, 5 = Chongón‐Colonche, 6 = Churute, 7 = Buenaventura, 8 = Achiotes‐El Faique, 9 = Cordillera Arañitas‐La Ceiba, 10 = Río Guajalito, 11 = Río Faisanes, 12 = Quebrada Zapadores. Approximate distribution of terrestrial South American ecoregions modified from Olson et al. (2010)

Anuran assemblages of 12 sites spanning four biogeographic units (hereafter BU) in western Ecuador: Equatorial Chocó (EC), Equatorial Tumbes (ET), Western Cordillera (WC), and Equatorial Pacific (EP) (modified from Olson et al., 2001; shapefile available at http://maps.tnc.org/gis_data.html and Morrone, 2014) were studied. For each BU, we compiled information for the following sites: (a) EC: Bilsa‐Mache Chindul and Río Canandé; (b) EP: Chongón Colonche, Jama Coaque, Ayampe‐Machalilla, and Churute; (c) ET: Achiotes‐El Faique, La Ceiba‐Cordillera Arañitas, and Buenaventura; (d) WC: Quebrada Zapadores, Río Faisanes, and Río Guajalito. In addition, we classify the 12 sites according to four forest types: dry, moist, montane, and transition (modified from Harling, 1979; Holdridge, Grenke, Hatheway, Liang, & Tosi, 1971) (Table 1; Figure 1).

**Table 1:** Biogeographic units, sites, geographic location, forest types, and environmental variables values

Biogeographic unit	Site	Coordinates	Forest type	Temp. (°C)	Precip. (ml)	Elevation (masl)	References
Equatorial Chocó	Río Canandé	00°31′47″N; 79°12′38″W	Moist	18.5	4,000	230–600	Morales, Yánez‐Muñoz, Meza‐Ramos, & Reyes‐Puig (2013)
	Bilsa – Mache Chindul	00°21′33″N; 79°42′02″W	Moist	22	1,900	300–750	Ortega‐Andrade et al. (2013); Jongsma et al. (2017)
	Chongón Colonche	01°52′00″S; 80°38′00″W	Transition	23.4	750	200–830	Amador & Martínez (2011); Present research
Equatorial Pacific	Churute	02°28′00″S; 79°43′20″W	Transition	25.5	900	30–900	Present research
	Machalilla – Ayampe	01°40′00″S; 80°43′00″W	Transition	24.5	350	40–400	Cisneros‐Heredia (2006); Morales and Altamirano‐Benavides (2013); Present research
	Jama Coaque	00°06′56″S; 80°06′35″W	Transition	25.1	1,200	500–840	Lynch, Maynard, Hamilton, and Burkart (2014)
	Buenaventura	03°38′47″S; 79°45′31″W	Transition	20	1,100	650–1,300	Yánez‐Muñoz et al. (2013)
Equatorial Tumbes	La Ceiba – C. Arañitas	04°24′13″S; 80°08′03″W	Dry	22.5	500	400–750	Díaz & Baus (2001)
	Achiotes – El Faique	04°07′00″S; 80°24′00″W	Dry	25.6	250	330–450	Almeida & Nogales (2005)
	Río Guajalito	00°13′00″S; 78°48′00″W	Montane	19	2,400	700–2,000	Yánez‐Muñoz & Morales (2013)
Western Cordillera	Río Faisanes	00°18′13″S; 78°52′09″W	Moist	17	1,900	1,300–1,400	Bustamante, Ron, & Coloma (2005)
	Quebrada Zapadores	00°13′59″S; 78°45′00″W	Montane	18	2,020	1,900–2,300	Bustamante et al. (2005)

Abbreviation: masl, meters above sea level.

### 2.2 Data collection

Analyses were based on species presence/absence matrices including sites and biogeographic units (Table S1). Each matrix was built using field data, data from the literature (e.g., Arteaga, Bustamante, & Guayasamin, 2013; Lynch & Duellman, 1997; Ortega‐Andrade, Bermingham, Aulestia, & Paucar, 2010), and data from amphibian collections at the Museum of Zoology of the Pontificia Universidad Católica del Ecuador (QCAZ, https://bioweb.bio; Ron, Merino‐Viteri, & Ortiz, 2019), the Museum of Natural History Gustavo Orcés of the Escuela Politécnica Nacional in Quito (MHNGO), and the Museum of the Faculty of Natural Sciences of the Universidad de Guayaquil (FCCNN‐UG). To compare biogeographic similarity and to complement the presence/absence records, we used checklists published in other locations in western Ecuador (Table 1). Species identification was performed using taxonomic keys and specialized literature, including the original descriptions of the species recorded (e.g., Coloma, 1995; Lynch & Duellman, 1997). Fieldwork was carried out in Machalilla‐Ayampe (between 2005 and 2006) (site 4), Chongón Colonche (between 2005 and 2015) (site 5) and Churute (between 2012 and 2015) (site 6) (Figure 1), using a sampling technique of free and unrestricted search of individuals called Complete Species Inventory, this method is the most efficient to obtain the largest number of individuals per species in less time (Rueda‐Almonacid, Castro, & Cortez, 2006).

### 2.3 Anuran assemblage alpha diversity and variation in species composition

The species richness (SR) was calculated for each site, the forest type and BU. Comparisons of *S* on BU's and forest type, respectively, were analyzed with linear regression models and perform ANOVA on the data; afterward, a Tukey test was used to determine which relationships were statistically significant. These analyses were performed in R 3.3.2 software (R Core Team, 2016). In order to address which species are shared and which are distinct in the anuran assemblage, we calculated the Jaccard index for pairs of sites. To represent the ordering relationships among sites per BU in a reduced and predetermined number of axes, an ordination analysis (nonmetric multidimensional scaling analysis, 2D‐NMDS) was performed on matrices constructed from Jaccard indices. To test for differences in species composition dissimilarity, a permutational multivariate analysis of variance (PERMANOVA, Anderson, 2001) was performed on the Jaccard similarity matrices using “Biogeographic Unit” as a fixed factor. The probability value (*p*_perm_) was calculated from a pseudo‐*F* distribution with 10,000 permutations. All analyses were performed using PERMANOVA+ in the PRIMER v6 statistical package (Clarke & Gorley, 2006). In order to evaluate the effects of geographical distance on dissimilarity in species composition (distance–decay relationship) and to have another measure if species turnover and beta diversity, we calculated the distance in km between all pairs of sites and plotted the calculated Jaccard index. The distance–decay relationship was quantified, in the data set the linear relation of Jaccard similarity to geographic distance (on both log‐transformed and original scales) was assessed using linear regression. This analysis was made using the *Vegan* package (Oksanen et al., 2016) in R 3.3.2 (R Core Team, 2016).

### 2.4 Phylogenetic diversity and phylogenetic structure

We used a phylogeny of anuran species present in the 12 sites, since some species did not have available sequences and other species have only been identified to the genus level, as is the case of several reported *Pristimantis*, we downloaded 70 sequences (761 base pairs in each sequence) of 16S mitochondrial gene available from Genbank (see Appendix S1). Phylogenetic relationships performed with 1,000 ultrafast bootstrap replicates and the most appropriate substitution model based on the Bayesian information criterion (BIC) were inferred using IQ‐tree (Nguyen, Schmidt, von Haeseler, & Minh, 2015) and ModelFinder (Kalyaanamoorthy, Minh, Wong, Haeseler, & Jermiin, 2017), respectively. The sequences were analyzed under the TIM2 + I + G model, and the maximum likelihood tree was saved as Newick format for analysis. With this phylogeny and the community presence/absence matrix as input, we performed phylogenetic metrics for each site. We calculated two diversity measures, first the phylogenetic diversity (PD) index, defined as the sum of branch lengths between root and tips for a community (Faith, 1992) for each site, and we compared PD with forest type and BU's; first, we fit a linear regression models, and then, we perform an ANOVA on the data; afterward, a Tukey test was used to determine which two variables had significant differences. These analyses were performed in R 3.3.2 software (R Core Team, 2016). And then, we calculated the standardized effect size of Faith's PD (SES_PD_) for all the sites. In order to assess how phylogenetically related are the average pair of species in a site, we use two indices proposed by Webb, Ackerly, McPeek, and Donoghue (2002) and modified by Kembel (2009) as measures of standardized effect size of phylogenetic community structure, SES_MPD_ and SES_MNTD_, which are the negations of Net Relatedness Index (NRI) and Nearest Taxon Index (NTI), respectively (Pearse, Jones, & Purvis, 2013). The community phylogenetic structure was calculated as follows:

$${\text{SES}}_{\text{MPD}}={\text{MPD}}_{\text{obs}}-{\text{MPD}}_{\text{rand\textbackslash{}\_mean}}/{\text{MPD}}_{\text{rand\textbackslash{}\_sd}}$$

$${\text{SES}}_{\text{MNTD}}={\text{MNTD}}_{\text{obs}}-{\text{MNTD}}_{\text{rand\textbackslash{}\_mean}}/{\text{MNTD}}_{\text{rand\textbackslash{}\_sd}}$$

MPD calculate the mean pairwise distance between all species in each site. On the other hand, MNTD calculates the mean nearest taxon distance, the mean of the branch lengths connecting each species to its closest relative (Webb, 2000). We use a null model of randomly shuffling tip labels across the tips of the phylogeny with 1,000 runs for each analysis (site). The reported *p*‐value was calculated with a two‐tailed test, thus, significance at the threshold α = 0.05 level is achieved when *p* ≤ .025 or *p* ≥ .975 (Cadotte & Davies, 2016). Positive SES values and high *p*‐values (*p* ≥ .975) indicate phylogenetic evenness and greater phylogenetic distance among co‐occurring species than expected, and negative SES values and low *p*‐values (*p* ≤ .025) indicate phylogenetic clustering and small phylogenetic distances among co‐occurring species than expected (Kembel et al., 2010). The analyses were performed with *PICANTE* package (Kembel et al., 2010) in R 3.3.2 software (R Core Team, 2016).

### 2.5 Effect of environment on anuran diversity

We evaluated the correlation between abiotic and biotic components. Correlation tests were performed between dissimilarity matrices (Bray–Curtis dissimilarity) of environmental variables (precipitation, temperature, and elevation between sites), and the inverse value of Jaccard similarity (J*dissim*). These models were calculated using the package Vegan in R (Oksanen et al., 2016) and following the recommendation by Legendre, Borcard, and Peres‐Neto (2005), Legendre, Fortin, and Borcard (2015) and Legendre and Legendre (1998). To detect multicollinearity of predictor variables, we used a statistic called the variance inflation factor (VIF) (Fox & Monette, 1992). The square root of the VIF indicates the degree to which the standard error is, comparing if a predictor variable was not correlated with other predictor variables in a model. As a general rule, $\sqrt{\mathrm{vif}}$ > 2 indicates a multicollinearity problem. VIF values were calculated with the *vif* function in the *car* package. Averages of temperature and precipitation from five recent years (2012–2016) were obtained from the Meteorological Service of Ecuador (http://www.serviciometeorologico.gob.ec), and elevation of the sites were obtained from Google Earth Pro (Google, Version 7.3.0.3832) (Table 1). Also, we evaluated if there was an effect of the three environmental variables previously mentioned (see Table 1) on *S* and PD (response variables), in order to select the best‐fit model that explains the maximum amount of variance, we created two multiple regression models to explain the two response variables, this was done in R 3.3.2 (R Core Team, 2016). To look for evidence of nonlinearity in the relationship between the dependent variable (*S* and PD) and the independent variables (precipitation, temperature, and elevation), we used *component plus residual plots* whit the *crPlots* function in the R package *car* (Fox & Weisberg, 2011). Similarly, we performed a global validation of linear model assumptions with the R package *gvlma* (Pena & Slate, 2006). To assess the relative importance, that is, the contribution of each of the predictor variables on the response variable in a multiple regression model; we use the R package *relaimpo* which provides several reasonable metrics, such as *lmg* that propose averaging sequential sums of squares over all orderings of regressors (Lindeman, Merenda, & Gold, 1980) for assessing relative importance (percent contribution) of each correlated predictor (regressor) in a linear model (Grömping, 2006).

## 3 RESULTS

### 3.1 Anuran assemblage alpha diversity and variation in species composition

**Figure 2:**
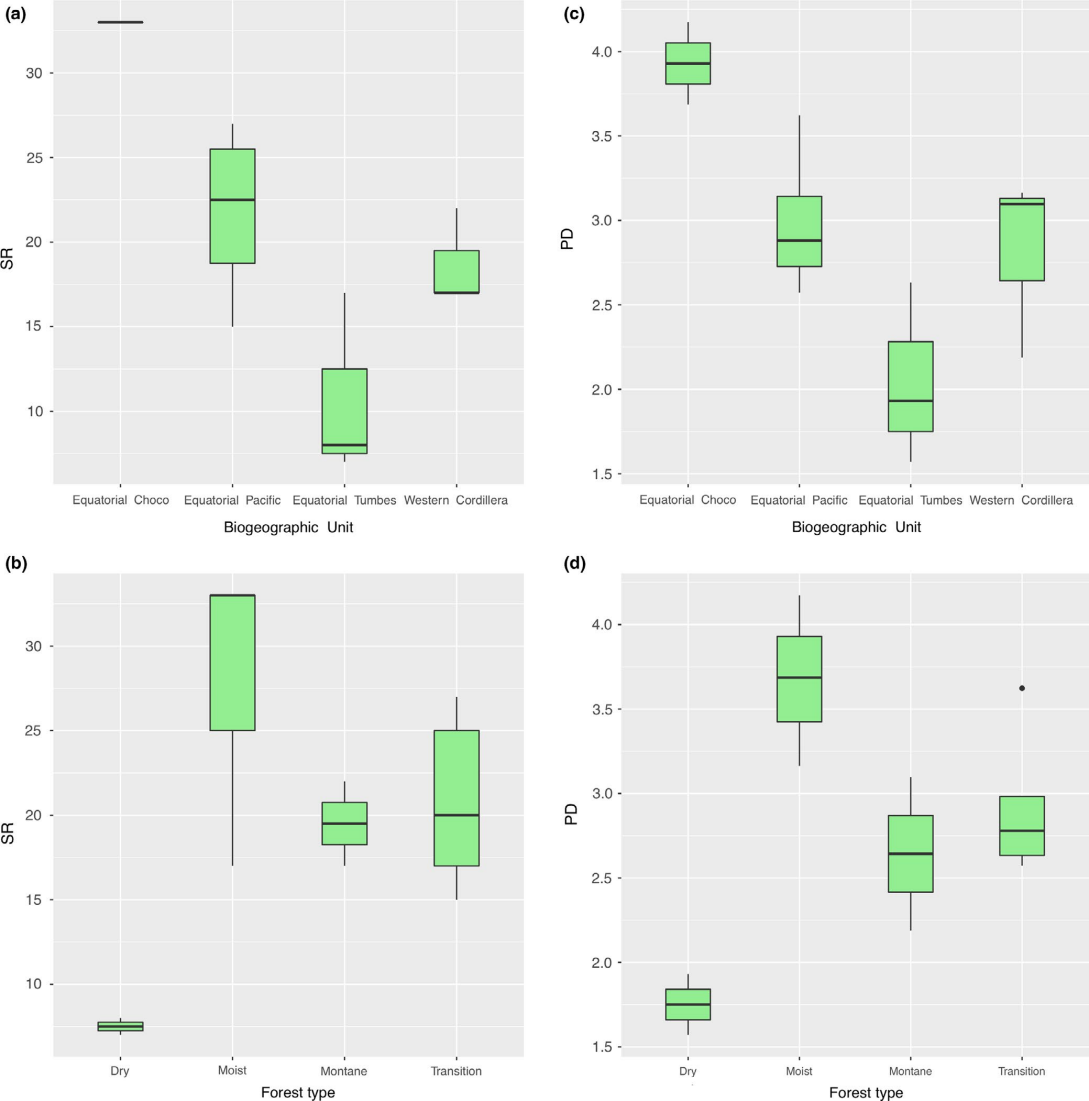
Boxplot showing species richness (SR) (a and b) and phylogenetic diversity (PD) (c and d) per biogeographic unit and forest type. Outliers are shown with black circles. Thick horizontal black lines indicate means

A total of 101 species of frogs were recorded (Table S1); they belong to the families Bufonidae (5 species), Centrolenidae (16), Ceratophryidae (1), Craugastoridae (41), Dendrobatidae (8), Hemiphractidae (2), Hylidae (14), Phyllomedusidae (2), Leptodactylidae (9), and Ranidae (2). The genus *Pristimantis* (Craugastoridae), with 37 species, had the highest species diversity. The most diverse sites were within the Equatorial Chocó (Río Canandé [site 1] and Bilsa‐Mache Chindul [site 2], each with 33 species). Within the Equatorial Pacific BU, the Chongón‐Colonche (site 5) had the highest *S* with 27 species. The sites with lowest *S* were in the Equatorial Tumbes BU; these sites included Cordillera Arañitas‐La Ceiba (site 9) and Achiotes‐El Faique (site 8) with seven and eight species, respectively. Regarding the BU's, Equatorial Chocó had the highest *S* with 44 species, and this was followed by the Equatorial Pacific (43 species), Western Cordillera (38 species), and Equatorial Tumbes (20 species) (Table 2; see also Figure 1 for site number). According to linear models, *S* differ significantly with the forest type (*F* (3,8) = 4.55, *p* = .038) (Figure 2), post hoc Tukey's HSD tests showed that only moist forests and dry forests had significant differences in species richness; all other comparisons were not significant. Further, strong significant differences were found in *S* for the different BU's (*F* (3,8) = 10.03, *p* = .004) (Figure 2), Tukey's tests showed significant differences of Equatorial Chocó with Equatorial Tumbes and Western Cordillera (Table S2).

**Table 2:** Values of species richness (SR), phylogenetic diversity (PD), phylogenetic distance (mean pairwise distance—MPD and mean nearest taxon distance—MNTD), phylogenetic structure calculated as standard effect size of MPD (SES_MPD_) and MNTD (SES_MNTD_) and standardized effect size of PD (SES_PD_). The *p*‐value reported is a two‐tailed test, where the significance level of 0.05 is achieved when *p* ≤ .025 or *p* ≥ .975. Number of amphibian species found in each biogeographic unit (are shown in parentheses)

Biogeographic units	Site	SR	PD	SES_PD_	MPD	SES_MPD_	*p*‐Value	MNTD	SES_MNTD_	*p*‐Value
Equatorial Pacific (43)	Chongon‐Colonche	27	91.36	0.486	0.527	−1.046	.135	0.259	0.174	.549
	Machalilla‐Ayampe	20	90.88	−0.316	0.551	0.109	.467	0.246	−0.867	.204
	Churute	15	93.02	−0.014	0.497	−1.893	.049	0.306	0.375	.632
	Jama‐Coaque	25	90.78	−0.630	0.537	−0.491	.267	0.229	−1.98	.124
Equatorial Chocó (44)	Bilsa‐Mache Chindul	33	91.60	−0.844	0.538	−0.606	.225	0.228	−0.486	.307
	Río Canandé	33	92.05	−0.259	0.53	−1.117	.136	0.232	0.036	.505
Western Cordillera (38)	Q. Zapadores	17	79.90	−2.710	0.534	−0.602	.221	0.194	−2.285	.015
	Río Faisanes	17	90.44	0.864	0.566	0.784	.784	0.306	0.947	.815
	Río Guajalito	22	80.81	−1.274	0.55	0.087	.441	0.227	−1.001	.165
Equatorial Tumbes (20)	La Ceiba‐C. Arañitas	7	93.65	−0.394	0.517	−0.882	.173	0.336	−0.277	.398
	Achiotes‐El Faique	8	97.62	0.827	0.535	−0.387	.275	0.411	1.184	.897
	Buenaventura	17	91.67	1.049	0.561	0.454	.632	0.347	1.106	.878

In relation to species composition variation in biogeographic units, the nMDS indicated that the sites form two main clusters of low similarity. The most distinct group included the Western Cordillera sites while the second group included the Chocó, Pacific, and Equatorial Tumbes sites (Figure S3). This result was complemented by high goodness of fit resulting from repeated optimization; the stress function of the nMDS was 0.069, which indicates that the scaling was properly adjusted. From a total of 44 species recorded in the Equatorial Chocó, only eight species were also present in W. Cordillera; similar variation occurs with Equatorial Pacific (43 species), and only eight were also in W. Cordillera. BU's that shared more species were E. Chocó–E. Pacific (18 species shared) and E. Pacific–E. Tumbes (16 species shared) (Table S3).

The results of the PERMANOVA analyses showed significant differences between sites (pseudo‐*F* (3, 8) = 3.278; *p* (perm) < .001) and between some of the BU's. Specifically, the Equatorial Pacific and Western Cordillera were significantly different (*t* = 20.21, *p* (MC) = .0288), and the *p* (MC) probability values of the comparison of Western Cordillera with Equatorial Tumbes and Equatorial Chocó suggested that there may be subtle differences between these units, *p* (MC) = .0558 and *p* (MC) = .0553, respectively (Table S5).

**Figure 3:**
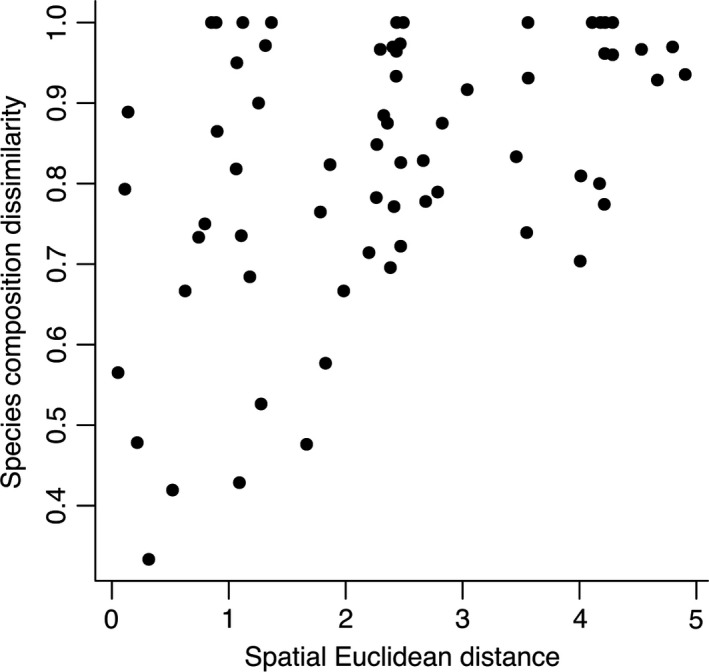
Distance–decay plot, the distance was measured in km between all pairs of sites (Spatial Euclidean distance) and plotted the calculated Jaccard index (Species composition dissimilarity)

When we evaluate the geographical distance with the Jaccard dissimilarity in species composition, we found a statistically significant positive correlation coefficient (*r* = .461, *p* < .001), indicating that there is a distance decay of similarity (communities far away from each other have more different species compositions) (Figure 3).

### 3.2 Phylogenetic diversity and phylogenetic structure

The relationship between SR and PD for the community data showed that PD is strongly correlated with SR (*p* < .001, *R^2^* = 0.86) (Figure S1). As expected, PD was found to be the highest in moist forests of Equatorial Chocó and the lowest in dry forest of Equatorial Tumbes. The highest SES_PD_ was found in Buenaventura (Equatorial Tumbes, transition forest), while the lowest SES_PD_ in Quebrada Zapadores (Western Cordillera, montane forest) (Table 2). There were differences in PD among different forest types (*F* (3,8) = 7.12, *p* = .012) and among different BU's as predictor (*F* (3,8) = 6.06, *p* = .019) (Figure 2). However, post hoc Tukey's HSD tests showed that forests comparisons do not have differences in PD, except moist–dry forests that were significant. On the other hand, Tukey's tests showed significant differences only Equatorial Tumbes‐Equatorial Chocó BU's, all other comparisons were not different (Table S2).

Phylogenetic structure of the anuran communities varied across the spatial extent of the study area (Table 2). There were no significant differences of SES_MPD_ when this standardized effect size was calculated for the different sites, forest types, and biogeographic units. Similar results were found with SES_MNTD_, there were no significant differences in the same three previous levels, only the site Q. Zapadores had a *p*‐value = .015 calculated with a two‐tailed test (Figure S2, Table 2).

### 3.3 Effect of environment on anuran diversity

**Figure 4:**
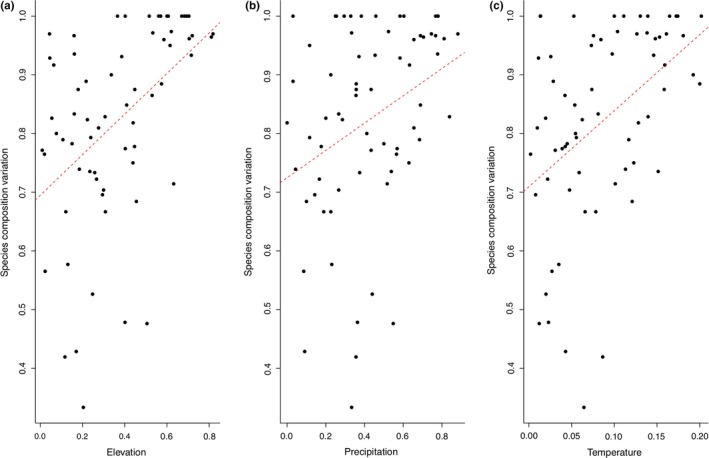
Correlations tests between dissimilarity (Species composition variation) and environmental variables (a) elevation, (b) precipitation, (c) temperature

**Figure 5:**
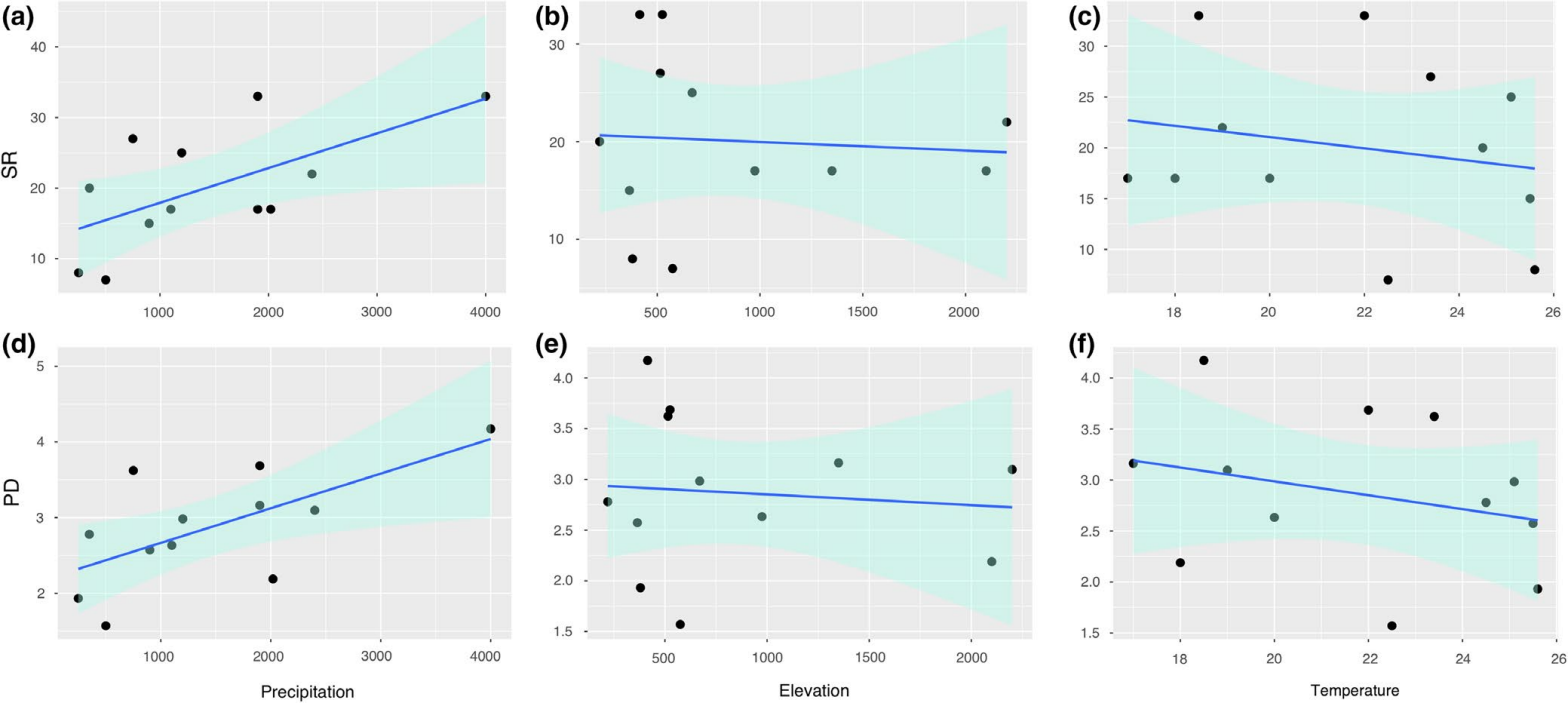
Scatterplots using a multiple regression model of environmental variables (precipitation, elevation, and temperature) values plotted against species richness values (SR, a–c) and phylogenetic diversity (PD, d–f)

We do not find multicollinearity evidence of variable predictors in this model, elevation $\sqrt{\mathrm{vif}}$ = 1.456, precipitation $\sqrt{\mathrm{vif}}$ = 1.504, temperature $\sqrt{\mathrm{vif}}$ = 1.956. Correlations between the value of Jaccard dissimilarity and the dissimilarity matrices of precipitation (*r* = .364, *p* = .004), temperature (*r* = .444, *p* < .001), and elevation (*r* = .470, *p* < .001) were positive and significant (Figure 4). We ran multiple regression linear models of *S* and PD using elevation, precipitation, and temperature as predictors (Figure 5). Components + Residuals plots and global validation of linear model (gvlma) confirm that linear model assumptions are true for all models (see Supporting information). According to the linear model, *S* did not differ significantly with elevation + precipitation + temperature (*F* (3,8) = 3.06, *p* = .092) (Table S4). However, a significant relationship was found between *S* and precipitation alone (*p* = .031), yet there was no significant effect of elevation and temperature on *S* (*p* > .05). PD was not significantly correlated with each of the three environmental variables examined (*F* (3,8) = 3.38, *p* = .075), as in *S* precipitation was significant (*p* = .046). However, the *p*‐value for elevation and temperature (0.324 and 0.885, respectively) is greater than the common alpha level of 0.05, which indicates that were not statistically significant (Table S4).

According the relative importance of three environment variables on PD and *S*, we use the method called *lmg* (Grömping, 2006). Precipitation had the highest relative importance or regressor contribution (*R* ^2^) on *S* (*lmg* = 79.8%) and PD (*lmg* = 72.5%) (Table 3). When comparing *lmg* with other methods to measure relative importance, similar results were obtained (Figure S4).

**Table 3:** Relative importance metrics for species richness and phylogenetic diversity. The lmg method (expressed as percentage of explained variance) was used in R package *relaimpo*

Dependent variable	Regressors	lmg value	Confidence interval (CI) (1,000 bootstrap replicates)^a^		
		%	0.95	Lower 0.95	Upper 0.95
Species richness	Elevation	0.0744	ABC	0.0214	0.4190
	Precipitation	0.7979	AB_	0.3238	0.8450
	Temperature	0.1277	_BC	0.0582	0.4200
Phylogenetic diversity	Elevation	0.1322	ABC	0.0274	0.5758
	Precipitation	0.7250	AB_	0.2616	0.8392
	Temperature	0.1427	_BC	0.0466	0.3684

a Letters (ABC) indicate the ranks included in the bootstrapped CIs. Rank bootstrap confidence intervals were obtained using the percentile method (bty = perc).

## 4 DISCUSSION

We found heterogeneity in the alpha, beta, and phylogenetic diversity among the four Ecuadorian biogeographic units. The Equatorial Chocó was the unit with the highest species richness, which can be mainly explained by climatic factors such as high average annual rainfall (2,000 mm; Sierra, Cerón, Palacios, & Valencia, 1999) and moisture throughout the year (Ortiz‐Yusty, Páez, & Zapata, 2013); this condition was corroborated in this work through regression models and analysis of relative importance. In contrast, Equatorial Tumbes is dominated by dry vegetation and is characterized by high seasonality where rainfall is <1,600 mm per year, and there are at least four to six dry months with rainfall <100 mm per month (Gentry, 1995; Mooney, Bullock, & Medina, 1995; Pennington, Lavin, & Oliveira‐Filho, 2009); likely as a result, amphibian richness was lower in Equatorial Tumbes. As in species richness, the differences in phylogenetic diversity in community assemblage of anurans are related to differences in precipitation; this is relevant to understand the turnover across different sites, forest types, and biogeographic units. The effect of precipitation may be due to the fact that alpha and phylogenetic diversity are inherently positively correlated, since a greater number of species almost always correlates with a greater genetic divergence summarized in a phylogeny (Cadotte & Davies, 2016; Venail et al., 2015).

The similarity/dissimilarity among the anuran communities studied here can be explained in part by environmental or climatic factors (Lynch & Suárez‐Mayorga, 2002). Here, we used ordination analysis to identify significant relationships between biological (e.g., number of species, species turnover and phylogenetic diversity) and environmental variables (e.g., temperature, precipitation) among sites. We found that biogeographic dissimilarity measured as species composition variation was significantly correlated with precipitation, temperature, and elevation (Figure 4). The Jaccard indices suggest that each of the biogeographic units (and some of the sites within) has characteristics that differentially influence species composition. Overall, low values of similarity, not exceeding 40% shared species, were found. It is noteworthy that out of the 101 species reported in this study, only four species were recorded in the four units: *Rhinella marina* (Bufonidae, nine sites), *Espadarana prosoblepon* (Centrolenidae, six sites), *Pristimantis achatinus* (Craugastoridae, eight sites), and *Boana pellucens* (Hylidae, six sites). Therefore, the largest proportion of recorded species are not shared when we move from one unit to another along a latitudinal or environmental gradient.

In this study, the genus *Pristimantis* (Craugastoridae) had the highest number of recorded species (36.6% of the total species recorded). Lynch and Duellman (1997) show that *Pristimantis* species from the lowlands of western Ecuador have wider distribution ranges than congenerics from the Andes; this could explain the high number of species of this genus recorded in the Western Cordillera (*Pristimantis* richness was much higher than that of other genera in the same unit).

According to the phylogenetic structure in the assembly of the communities, most of the communities are phylogenetically grouped (e.g., La Ceiba‐Cordillera Arañitas, Quebrada Zapadores, Bilsa‐Mache Chindul, Jama‐Coaque), however, no significant differences were found. Only two sites (Buenaventura and Río Faisanes) presented are high phylogenetically overdispersal or a greater phylogenetic distance between coexisting species than expected. These results could be explained given that the community assemblages consist mainly of species that have diverged relatively recently.

Because species richness and distribution patterns at local scales are the result of complex biotic and abiotic interactions at many spatial and temporal scales (Wisz et al., 2013), there is no single cause of these patterns. On the other hand, environmental factors such as precipitation or elevation can influence ecological processes in organisms, and therefore their capacity for dispersion and persistence in different environments (Brown & Lomolino, 1998). By analyzing precipitation, temperature, and elevation, we have sought to widen our inference of the factors affecting the distribution of amphibians in western Ecuador. Others have found that the species richness of amphibians is influenced by factors such as temperature, geography, and precipitation (e.g., Ortiz‐Yusty et al., 2013; Soares & Brito, 2007). Nonetheless, these factors are not the only studied, other studies have also found that anuran diversity has been determined as a response to either different types of vegetation, distance to water bodies or environmental heterogeneity (e.g., Goncalves, Crivellari, & Conte, 2015; Ribeiro et al., 2017). Here, from the regression analysis, we also found that precipitation could have a strong effect on the diversity of amphibians (SR‐PD; Figure 5, Figure S4).

The results of this work may suggest that Equatorial Pacific would act as a transition zone between Equatorial Chocó (wet/moist northern forests) and Equatorial Tumbes (dry southern forests) (see Figure 1) in terms of anuran species composition. Overall, our results support this suggestion, as has been previously defined for the area (Valverde, 1991; Yánez‐Muñoz, Morales, Reyes‐Puig, & Meza‐Ramos, 2013). These three biogeographic units mentioned above, could be characterized by high species turnover, which would follow a latitudinal gradient. For example, among these three units the species composition of some dendrobatids varies latitudinal and ecologically (see Coloma, 1995; Grant et al., 2006, 2017; Santos et al., 2009; Tarvin, Powell, Santos, Ron, & Cannatella, 2017, for distribution data). As in the case of replacement, in the biogeographic units mentioned above, of three *Epipedobates* species: *E. boulengeri* (Chocó)–*E. machalilla* (Transition Zone)–*E. anthonyi* (Tumbes), and three *Hyloxalus* species: *H. awa* (Chocó)–*H. infraguttatus* (Transition Zone)–*H. elachyhistus*.

We conclude that environmental factors such as precipitation, elevation, and temperature could affect the diversity of anurans in Western Ecuador. For example, the composition of anuro‐fauna in the forests of the Western Cordillera, sites that present low temperatures on average, is markedly different from the composition found in three other biogeographic units, presenting a high species richness but belonging to a few taxonomic groups (e.g., rainfrogs of genus *Pristimantis* or glassfrogs of family Centrolenidae). On the other hand, the high rainfall in the Ecuadorian Chocó lead to the sites in this biogeographic unit maintain a constant humidity throughout the year, which make available numerous ideal microhabitats for the persistence of several amphibian species distributed in different clades within a phylogenetic tree.

Finally, these ecosystems in the coast and western Andes of Ecuador have already been categorized as high priority areas for conservation and as high exposure risk zones (Cuesta et al., 2017; Sierra, Campos, & Chamberlin, 2002). Furthermore, given the high phylogenetic diversity of amphibians and even because most of the sites in this study do not have a formal declaration of forest protection, which could allow these forests to be considered as conservation areas of biodiversity (see also Arteaga et al., 2013; Cuesta et al., 2017; Lessmann et al., 2014).

## CONFLICT OF INTEREST

None declared.

## AUTHOR CONTRIBUTIONS

LA and JMG conceived the study concepts; LA, JMG, and MS‐G designed the methodology; LA collected and analyzed the data; LA, MS‐G, and JMG led the writing of the manuscript. All authors contributed critically to the drafts and provided final approval for publication.

## Data Availability

R code for statistical, ecological, and phylogenetic analyses, as well as the input files are available on Figshare, https://doi.org/10.6084/m9.figshare.7749320.
