# Supplementary figures and images for: Integrating alpha, beta, and phylogenetic diversity to understand anuran fauna along environmental gradients of tropical forests in western Ecuador
Source: Ecol Evol. 2019 Sep 12;9(19):11040–52. doi: 10.1002/ece3.5593 (PMC6802013; doi:10.1002/ece3.5593)

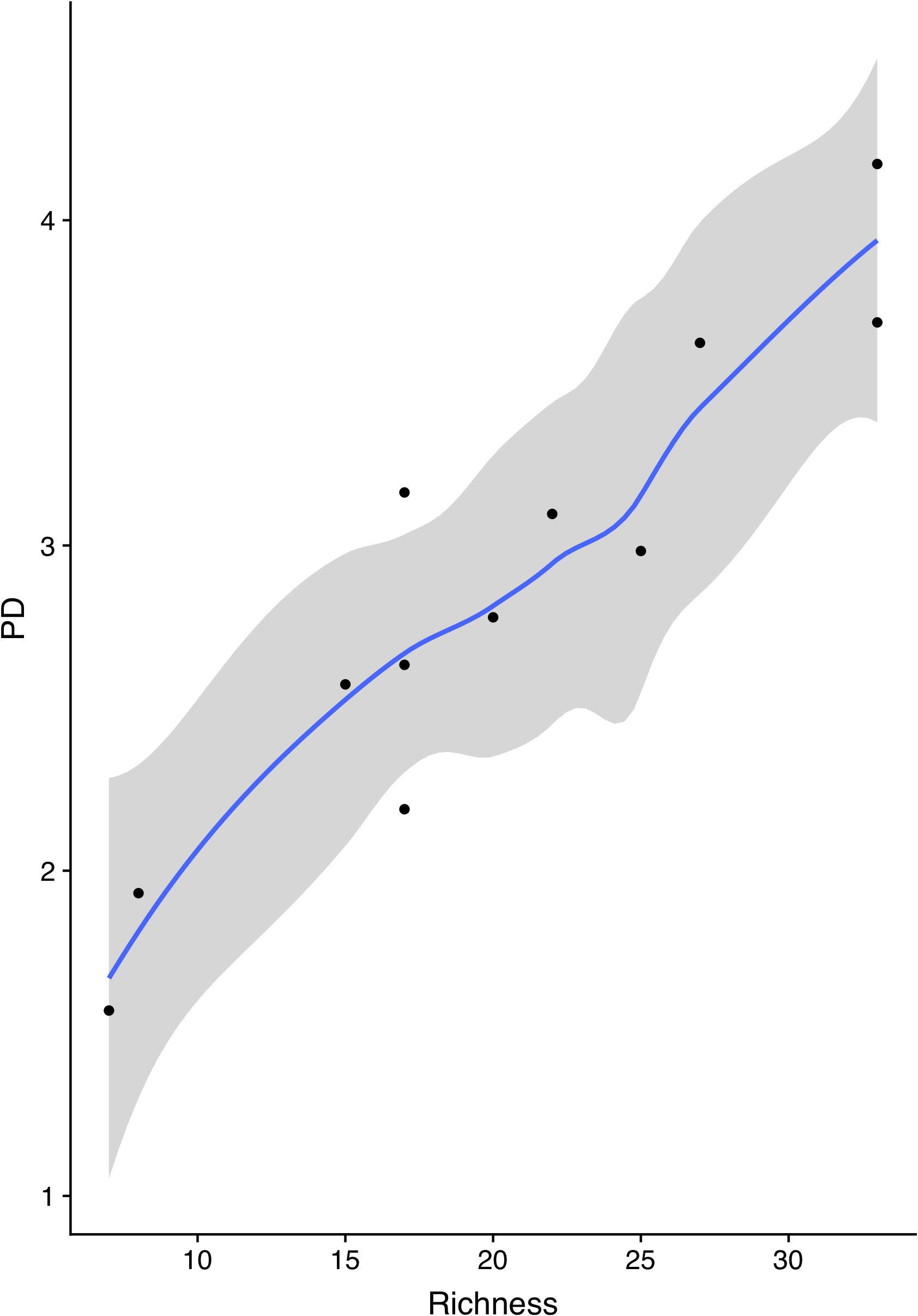

Supplement: Supplementary file 1 [file ECE3-9-11040-s001.png]

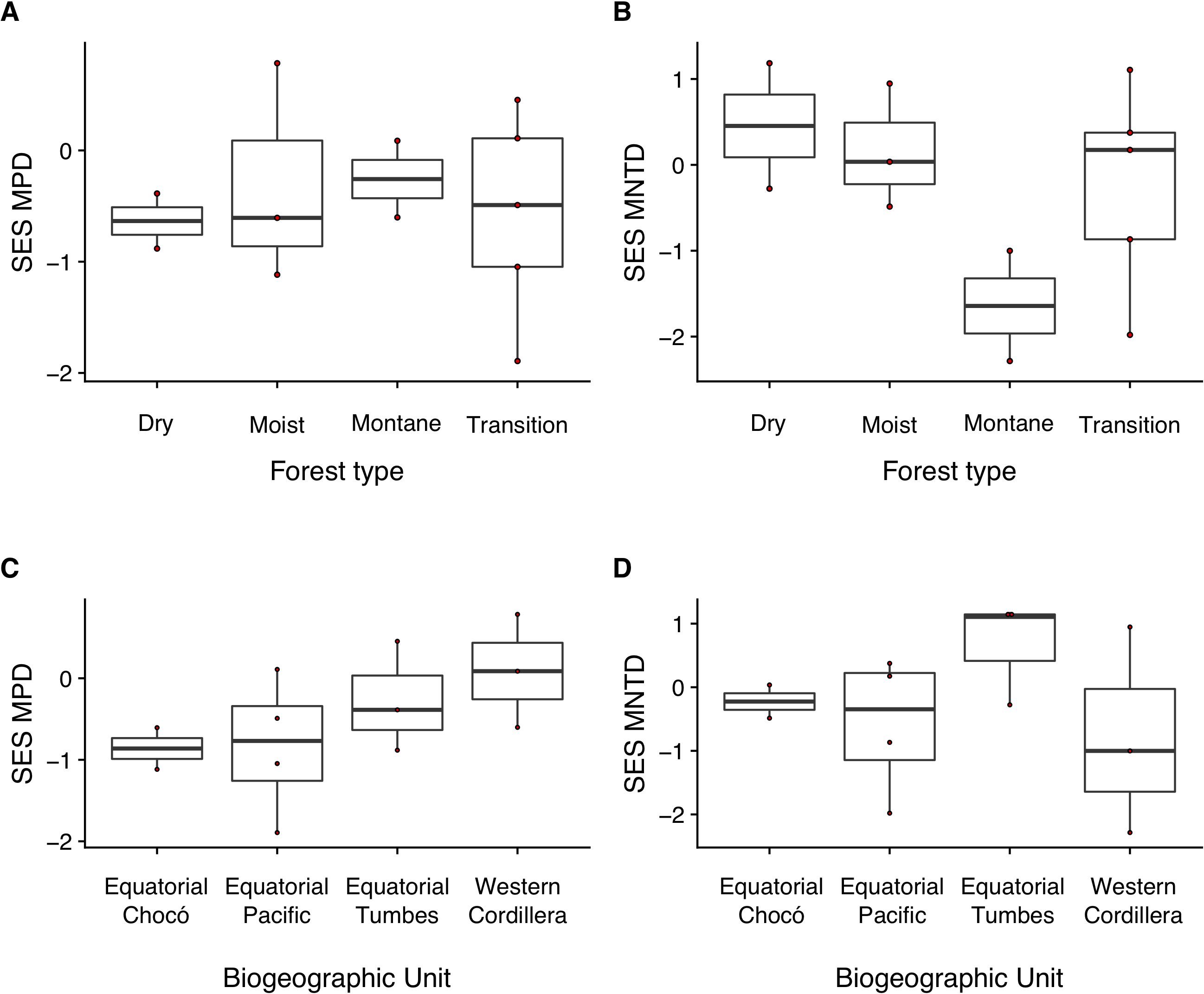

Supplement: Supplementary file 2 [file ECE3-9-11040-s002.png]

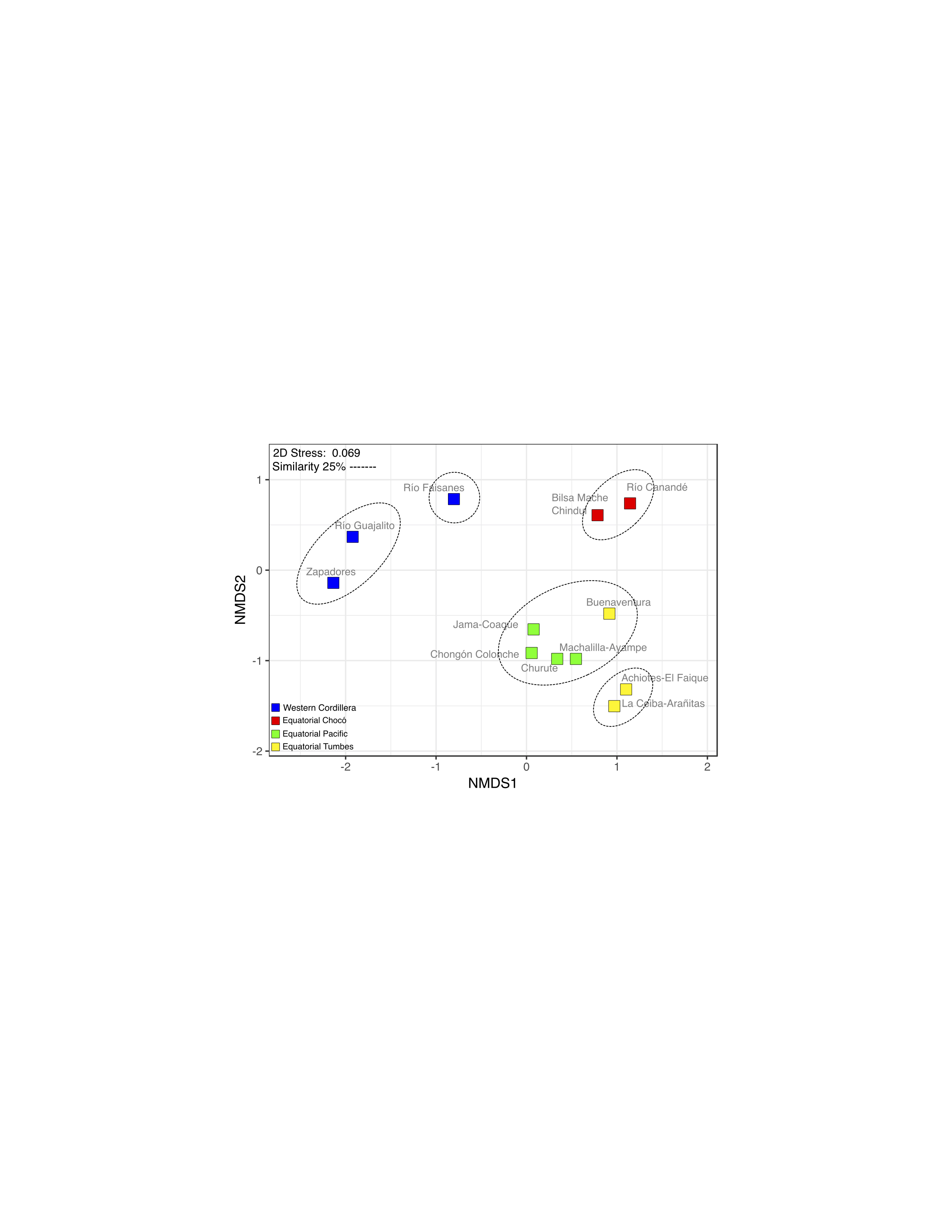

Supplement: Supplementary file 3 [file ECE3-9-11040-s003.png]

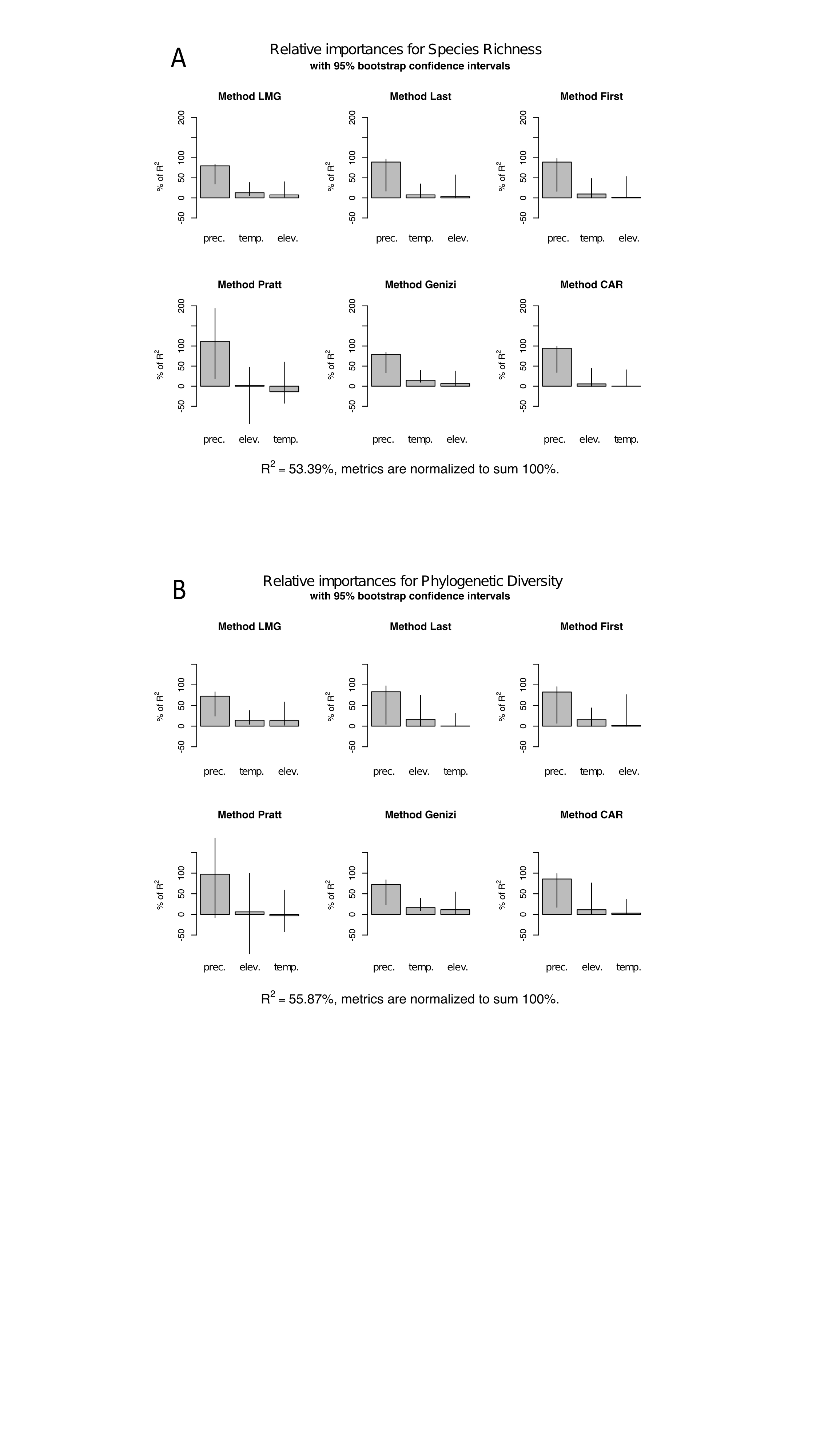

Supplement: Supplementary file 4 [file ECE3-9-11040-s004.png]
