# Supplementary material for: Integrating alpha, beta, and phylogenetic diversity to understand anuran fauna along environmental gradients of tropical forests in western Ecuador
Source: Ecol Evol. 2019 Sep 12;9(19):11040–52. doi: 10.1002/ece3.5593 (PMC6802013; doi:10.1002/ece3.5593)
Supplement: Supplementary file 5 [file ECE3-9-11040-s005.docx]

**SUPPLEMENTARY MATERIAL**

**Table S1**. Amphibian species in 12 registered sites from four biogeographic units in Western Ecuador. Acronyms: CchC= Cordillera Chongon Colonche; MA-AY= Machalilla-Ayampe; B-MCh= Bilsa-Mache Chindul; Ja-CO= Jama Coaque; Q. Zap. = Quebrada Zapadores; CA-LC= Cordillera Arañitas-La Ceiba; Ach-EF= Achiotes-El Faique; Bvent= Buenaventura.

|  | **Equatorial Pacific** | | | | **Equatorial Choco** | | **Western Andes** | | | **Equatorial Tumbes** | | |
| --- | --- | --- | --- | --- | --- | --- | --- | --- | --- | --- | --- | --- |
| **Species** | **CChC** | **MA-AY** | **Churute** | **Ja-Co** | **B-MCh** | **Río Canande** | **Q. Zap.** | **Río Faisanes** | **Río Guajalito** | **CA-LC** | **Ach-EF** | **Bvent** |
| *Atelopus longirostris* |  |  |  |  |  |  |  | 1 | 1 |  |  |  |
| *Atelopus mindoensis* |  |  |  |  |  |  | 1 |  |  |  |  |  |
| *Rhaebo haematiticus* |  |  |  |  | 1 | 1 |  |  |  |  |  |  |
| *Rhinella alata* |  |  |  |  | 1 | 1 |  |  |  |  |  |  |
| *Rhinella marina* | 1 | 1 | 1 | 1 | 1 |  |  |  | 1 | 1 | 1 | 1 |
| *Centrolene geckoideum* |  |  |  |  |  |  | 1 |  | 1 |  |  |  |
| *Centrolene heloderma* |  |  |  |  |  |  | 1 |  |  |  |  |  |
| *Centrolene lynchi* |  |  |  |  |  |  |  | 1 | 1 |  |  |  |
| *Cochranella mache* |  |  |  | 1 | 1 | 1 |  |  |  |  |  |  |
| *Espadarana prosoblepon* | 1 |  |  | 1 | 1 | 1 |  | 1 |  |  |  | 1 |
| *Hyalinobatrachium aureoguttatum* |  |  |  |  |  | 1 |  |  |  |  |  |  |
| *Hyalinobatrachium fleischmanni* |  |  |  | 1 | 1 |  |  |  |  |  |  |  |
| *Hyalinobatrachium valerioi* |  |  |  |  | 1 |  |  | 1 |  |  |  |  |
| *Nymphargus grandisonae* |  |  |  |  |  |  | 1 | 1 | 1 |  |  |  |
| *Nymphargus griffithsi* |  |  |  |  |  |  | 1 |  | 1 |  |  |  |
| *Nymphargus buenaventura* |  |  |  |  |  |  |  |  |  |  |  | 1 |
| *Sachatamia orejuela* |  |  |  |  |  | 1 |  |  |  |  |  |  |
| *Sachatamia albomaculata* |  |  |  |  | 1 | 1 |  |  |  |  |  |  |
| *Sachatamia ilex* |  |  |  |  |  | 1 |  |  |  |  |  |  |
| *Teratohyla pulverata* |  |  |  |  | 1 |  |  |  |  |  |  |  |
| *Teratohyla spinosa* |  |  |  |  | 1 | 1 |  |  |  |  |  |  |
| *Noblella coloma* |  |  |  |  |  |  |  |  | 1 |  |  |  |
| *Craugastor longirostris* | 1 |  |  |  | 1 | 1 |  |  |  |  |  |  |
| *Pristimantis achatinus* | 1 | 1 | 1 | 1 | 1 | 1 |  | 1 |  |  |  | 1 |
| *Pristimantis appendiculatus* |  |  |  |  |  |  | 1 |  | 1 |  |  |  |
| *Pristimantis calcarulatus* |  |  |  |  |  |  | 1 | 1 | 1 |  |  |  |
| *Pristimantis crenungis* |  |  |  |  |  |  |  | 1 |  |  |  |  |
| *Pristimantis crucifer* |  |  |  |  |  |  |  |  | 1 |  |  |  |
| *Pristimantis degener* |  |  |  |  |  | 1 |  |  |  |  |  |  |
| *Pristimantis dissimulatus* |  |  |  |  |  |  | 1 |  |  |  |  |  |
| *Pristimantis duellmani* |  |  |  |  |  |  | 1 |  |  |  |  |  |
| *Pristimantis eremitus* |  |  |  |  |  |  |  |  | 1 |  |  |  |
| *Pristimantis esmeraldas* |  |  |  |  |  | 1 |  |  |  |  |  |  |
| *Pristimantis eugeniae* |  |  |  |  |  |  | 1 |  | 1 |  |  |  |
| *Pristimantis floridus* |  |  |  |  |  |  | 1 |  |  |  |  |  |
| *Pristimantis labiosus* |  |  |  |  |  | 1 |  |  |  |  |  |  |
| *Pristimantis latidiscus* |  |  |  | 1 | 1 | 1 |  |  |  |  |  |  |
| *Pristimantis lymani* |  |  |  |  |  |  |  |  |  | 1 | 1 |  |
| *Pristimantis luteolateralis* |  |  |  |  |  |  |  | 1 |  |  |  |  |
| *Pristimantis muricatus* |  |  |  | 1 | 1 |  |  |  |  |  |  |  |
| *Pristimantis parvillus* |  |  |  |  | 1 |  |  |  | 1 |  |  |  |
| *Pristimantis quinquagesimus* |  |  |  |  |  |  |  |  | 1 |  |  |  |
| *Pristimantis rosadoi* |  |  |  |  | 1 | 1 |  |  |  |  |  |  |
| *Pristimantis sobetes* |  |  |  |  |  |  | 1 |  | 1 |  |  |  |
| *Pristimantis subsigillatus* |  | 1 |  | 1 | 1 | 1 |  |  |  |  |  | 1 |
| *Pristimantis unistrigatus* |  |  |  |  |  |  |  |  | 1 |  |  |  |
| *Pristimantis verecundus* |  |  |  |  |  |  |  |  | 1 |  |  |  |
| *Pristimantis walkeri* | 1 | 1 |  | 1 | 1 | 1 |  |  |  |  |  | 1 |
| *Pristimantis w-nigrum* |  |  |  |  |  |  | 1 |  | 1 |  |  |  |
| *Pristimantis* cf. *nyctophylax* | 1 |  |  |  |  |  |  | 1 |  |  |  |  |
| *Pristimantis* cf. *phoxocephalus* |  | 1 |  |  |  |  | 1 |  |  |  |  |  |
| *Pristimantis* sp1. | 1 |  |  |  |  |  |  |  |  |  |  |  |
| *Pristimantis* sp2. | 1 |  |  |  |  |  |  |  |  |  |  |  |
| *Pristimantis* sp3. | 1 |  |  |  |  |  |  |  |  |  |  |  |
| *Pristimantis* sp4. |  | 1 |  |  |  |  |  |  |  |  |  |  |
| *Pristimantis* sp5*.* |  |  |  |  | 1 |  |  |  |  |  |  |  |
| *Pristimantis* sp6. |  |  |  | 1 |  |  |  |  |  |  |  |  |
| *Pristimantis* sp7. |  |  |  | 1 |  |  |  |  |  |  |  |  |
| *Pristimantis* sp. (grp. unistrigatus) |  |  |  |  |  |  |  |  |  |  |  | 1 |
| *Pristimantis* sp. (grp. rubicundus) |  |  |  |  |  |  |  |  |  |  |  | 1 |
| *Barycholos pulcher* | 1 | 1 | 1 | 1 |  |  |  |  |  |  |  | 1 |
| *Strabomantis necerus* |  |  |  |  |  |  |  | 1 |  |  |  |  |
| *Colostethus* sp*.* |  |  |  |  | 1 |  |  |  |  |  |  |  |
| *Hyloxalus awa* |  |  |  | 1 | 1 | 1 |  | 1 |  |  |  |  |
| *Hyloxalus infraguttatus* complex | 1 | 1 | 1 |  |  |  |  |  |  |  | 1 | 1 |
| *Epipedobates anthonyi* |  |  |  |  |  |  |  |  |  | 1 | 1 | 1 |
| *Epipedobates boulengeri* |  |  |  |  | 1 | 1 |  |  |  |  |  |  |
| *Epipedobates darwinwallacei* |  |  |  |  |  |  |  | 1 |  |  |  |  |
| *Epipedobates machalilla* | 1 | 1 | 1 | 1 |  |  |  |  |  |  |  |  |
| *Oophaga sylvatica* |  |  |  |  | 1 | 1 |  |  |  |  |  |  |
| *Diasporus gularis* |  |  |  |  |  | 1 |  |  |  |  |  |  |
| *Gastrotheca guentheri* |  |  |  |  |  |  | 1 |  | 1 |  |  |  |
| *Gastrotheca plumbea* |  |  |  |  |  |  | 1 |  | 1 |  |  |  |
| *Agalychnis psilopygion* |  |  |  | 1 |  |  |  |  |  |  |  |  |
| *Agalychnis spurelli* |  | 1 |  |  | 1 | 1 |  |  |  |  |  |  |
| *Dendropsophus carnifex* |  |  |  |  |  |  | 1 | 1 | 1 |  |  |  |
| *Dendropsophus ebraccatus* |  |  |  |  |  | 1 |  |  |  |  |  |  |
| *Ecnomiohyla* sp. (cf. *phantasmagoria*) |  |  |  |  | 1 |  |  |  |  |  |  |  |
| *Boana pellucens* | 1 |  | 1 |  | 1 | 1 |  | 1 |  |  |  | 1 |
| *Boana picturata* |  |  |  |  | 1 | 1 |  | 1 |  |  |  |  |
| *Boana boans* | 1 |  |  |  | 1 | 1 |  |  |  |  |  | 1 |
| *Boana rosenbergi* | 1 |  |  | 1 | 1 |  |  |  |  |  |  |  |
| *Scinax quinquefasciatus* | 1 | 1 | 1 | 1 | 1 |  |  |  |  |  |  |  |
| *Scinax sugillatus* |  | 1 |  |  |  |  |  |  |  |  |  |  |
| *Smilisca phaeota* | 1 |  |  | 1 | 1 | 1 |  |  |  |  |  | 1 |
| *Trachycephalus jordani* | 1 | 1 | 1 | 1 |  |  |  |  |  |  | 1 |  |
| *Trachycephalus quadrangulum* | 1 | 1 | 1 | 1 |  |  |  |  |  |  |  |  |
| *Hyloscirtus alytolylax* |  |  | 1 |  |  |  |  | 1 | 1 |  |  | 1 |
| *Hyloscirtus palmeri* |  |  |  |  |  | 1 |  |  |  |  |  |  |
| *Engystomops* *guayaco* | 1 |  | 1 |  |  |  |  |  |  |  |  |  |
| *Engystomops montubio* | 1 | 1 |  | 1 |  |  |  |  |  |  |  |  |
| *Engystomops pustulatus* | 1 | 1 | 1 | 1 |  |  |  |  |  | 1 | 1 |  |
| *Engystomops randi* | 1 |  | 1 |  |  |  |  |  |  |  |  |  |
| *Leptodactylus labrosus* | 1 | 1 | 1 | 1 | 1 | 1 |  |  |  | 1 |  | 1 |
| *Leptodactylus melanonotus* |  | 1 |  |  |  | 1 |  |  |  |  |  |  |
| *Leptodactylus peritoaktites* |  |  |  | 1 |  |  |  |  |  |  |  |  |
| *Leptodactylus rhodomerus* |  |  |  |  | 1 | 1 |  |  |  |  |  |  |
| *Leptodactylus ventrimaculatus* | 1 | 1 |  | 1 |  | 1 |  |  |  | 1 | 1 |  |
| *Lithobates bwana* |  |  |  |  |  |  |  |  |  | 1 | 1 | 1 |
| *Lithobates vaillanti* | 1 |  |  |  |  |  |  |  |  |  |  |  |
| *Ceratophrys stolzmanni* | 1 | 1 | 1 |  |  |  |  |  |  |  |  |  |
| **Total species** | 27 | 20 | 15 | 25 | 33 | 33 | 17 | 17 | 22 | 7 | 8 | 17 |

**Table S2.** Regression, One-way ANOVA and Tukey’s post hoc tests used to examine whether the diversity metrics differ in forest types and biogeographic units.

| **Formula** |  | **Estimate** | **Std. Error** | **t-value** | **p-value** |  |
| --- | --- | --- | --- | --- | --- | --- |
|  | Dry forest | 7.500 | 4.246 | 1.766 | 0.115 |  |
|  | Moist forest | 20.167 | 5.482 | 3.679 | 0.006 | ** |
| lm(Species Richness ~ Forest type) | Montane forest | 12.000 | 6.005 | 1.998 | 0.081 | . |
|  | Transition forest | 13.300 | 5.024 | 2.647 | 0.029 | * |
|  | Multiple R-squared: 0.6306, Adjusted R-squared: 0.4921 | | | | |  |
|  |  |  |  |  |  |  |
| **Analysis of Variance Table (aov)** |  |  |  |  |  |  |
|  | **Df** | **Sum Sq** | **Mean Sq** | **F-value** | **p-value** |  |
| Forest type | 3 | 492.45 | 164.150 | 4.552 | 0.038 | * |
| Residuals | 8 | 288.47 | 36.058 |  |  |  |

**Tukey multiple comparisons of means - 95% family-wise confidence level**

Fit: aov(Species Richness ~ Forest type)

diff lwr upr p adj

Moist-Dry 20.166667 2.612457 37.720876 0.0257525

Montane-Dry 12.000000 -7.229673 31.229673 0.2646054

Transition-Dry 13.300000 -2.788698 29.388698 0.1093974

Montane-Moist -8.166667 -25.720876 9.387543 0.4853193

Transition-Moist -6.866667 -20.910034 7.176701 0.4467186

Transition-Montane 1.300000 -14.788698 17.388698 0.9934429

| **Formula** |  | **Estimate** | **Std. Error** | **t-value** | **p-value** |  |
| --- | --- | --- | --- | --- | --- | --- |
|  | Equatorial Chocó | 33.000 | 3.202 | 10.305 | 6.78e-06 | *** |
|  | Equatorial Pacific | -11.250 | 3.922 | -2.868 | 0.021 | * |
| lm(Species Richness ~ Biogeographic Unit) | Equatorial Tumbes | -22.333 | 4.134 | -5.402 | 0.001 | *** |
|  | Western Cordillera | -14.333 | 4.134 | -3.467 | 0.008 | ** |
|  | Multiple R-squared: 0.7899, Adjusted R-squared: 0.7111 | | | | |  |
|  |  |  |  |  |  |  |
| **Analysis of Variance Table (aov)** |  |  |  |  |  |  |
|  | **Df** | **Sum Sq** | **Mean Sq** | **F-value** | **p-value** |  |
| Biogeographic Unit | 3 | 616.83 | 205.61 | 10.025 | 0.004 | ** |
| Residuals | 8 | 164.08 | 20.51 |  |  |  |

**Tukey multiple comparisons of means - 95% family-wise confidence level**

Fit: aov(formula = Species Richness ~ Biogeographic Unit)

diff lwr upr p adj

Equatorial Pacific-Equatorial Choco -11.250000 -23.809922 1.309921975 0.0801100

Equatorial Tumbes-Equatorial Choco -22.333333 -35.572654 -9.094013108 0.0028618

Western Cordillera-Equatorial Choco -14.333333 -27.572654 -1.094013108 0.0345224

Equatorial Tumbes-Equatorial Pacific -11.083333 -22.160143 -0.006523322 0.0498675

Western Cordillera-Equatorial Pacific -3.083333 -14.160143 7.993476678 0.8096377

Western Cordillera-Equatorial Tumbes 8.000000 -3.841608 19.841608000 0.2129600

| **Formula** |  | **Estimate** | **Std. Error** | **t-value** | **p-value** |  |
| --- | --- | --- | --- | --- | --- | --- |
|  | Dry forest | 1.751 | 0.327 | 5.357 | 0.001 | *** |
|  | Moist forest | 1.924 | 0.422 | 4.560 | 0.002 | ** |
| lm(Phylogenetic Diversity ~ Forest type) | Montane forest | 0.892 | 0.462 | 1.931 | 0.090 | . |
|  | Transition forest | 1.168 | 0.387 | 3.020 | 0.017 | * |
|  | Multiple R-squared: 0.7275, Adjusted R-squared: 0.6253 | | | | |  |
|  |  |  |  |  |  |  |
| **Analysis of Variance Table (aov)** |  |  |  |  |  |  |
|  | **Df** | **Sum Sq** | **Mean Sq** | **F-value** | **p-value** |  |
| Forest type | 3 | 4.560 | 1.520 | 7.118 | 0.012 | * |
| Residuals | 8 | 1.708 | 0.214 |  |  |  |

**Tukey multiple comparisons of means - 95% family-wise confidence level**

Fit: aov(formula = Phylogenetic Diversity ~ Forest type)

diff lwr upr p adj

Moist-Dry 1.9235 0.57255300 3.2744470 0.0080087

Transition-Dry 1.1677 -0.07046338 2.4058634 0.0646515

Montane-Dry 0.8925 -0.58738830 2.3723883 0.2883691

Montane-Moist -1.0310 -2.38194700 0.3199470 0.1452980

Transition-Moist -0.7558 -1.83655760 0.3249576 0.1922675

Transition-Montane 0.2752 -0.96296338 1.5133634 0.8897089

| **Formula** |  | **Estimate** | **Std. Error** | **t-value** | **p-value** |  |
| --- | --- | --- | --- | --- | --- | --- |
|  | Equatorial Chocó | 3.930 | 0.350 | 11.359 | 3.25e-06 | *** |
|  | Equatorial Pacific | -0.940 | 0.424 | -2.219 | 0.057 | . |
| lm(Phylogenetic Diversity ~ Biogeographic Unit) | Equatorial Tumbes | -1.885 | 0.447 | -4.221 | 0.003 | ** |
|  | Western Cordillera | -1.113 | 0.447 | -2.493 | 0.037 | * |
|  | Multiple R-squared: 0.6946, Adjusted R-squared: 0.5801 | | | | |  |
|  |  |  |  |  |  |  |
| **Analysis of Variance Table (aov)** |  |  |  |  |  |  |
|  | **Df** | **Sum Sq** | **Mean Sq** | **F-value** | **p-value** |  |
| Biogeographic Unit | 3 | 4.354 | 1.451 | 6.065 | 0.019 | * |
| Residuals | 8 | 1.915 | 0.239 |  |  |  |

**Tukey multiple comparisons of means - 95% family-wise confidence level**

Fit: aov(formula = Phylogenetic Diversity ~ Biogeographic Unit)

diff lwr upr p adj

Equatorial_Pacific-Equatorial_Choco -0.9400000 -2.2967291 0.4167291 0.1977325

Equatorial_Tumbes-Equatorial_Choco -1.8848333 -3.3149514 -0.4547153 0.0124239

Western_Cordillera-Equatorial_Choco -1.1131667 -2.5432847 0.3169514 0.1358021

Equatorial_Tumbes-Equatorial_Pacific -0.9448333 -2.1413560 0.2516893 0.1291418

Western_Cordillera-Equatorial_Pacific -0.1731667 -1.3696893 1.0233560 0.9650066

Western_Cordillera-Equatorial_Tumbes 0.7716667 -0.5074698 2.0508032 0.2881515

**Table S3.** Amphibian species shared among biogeographic units (bold above diagonal) and number of amphibian species found in each biogeographic unit (underlined in the diagonal). The intensity of the gray coloration of the blocks under the diagonal represents the proportion of species shared among biogeographic units.

|  | **Equatorial Chocó** | **Equatorial Pacific** | **Equatorial Tumbes** | **Western Andes** |
| --- | --- | --- | --- | --- |
| **Equatorial Chocó** | 44 | **18** | **10** | **8** |
| **Equatorial Pacific** |  | 43 | **16** | **8** |
| **Equatorial Tumbes** |  |  | 20 | **5** |
| **Western Andes** |  |  |  | 38 |

| **Table S4.** Results of partial regression coefficients, values of the statistic (t value) and their associated significance value (p value), for the multiple regression models including species richness and phylogenetic diversity as response variables. | | | | | | | |  |  |  |
| --- | --- | --- | --- | --- | --- | --- | --- | --- | --- | --- |
| **Formula** |  | ***Estimate*** | | ***Std. Error*** | ***t value*** | ***p value*** |  |  |  |  |
|  | Intercept | -9.76228 | | 33.12337 | -0.29 | 0.776 |  |  |  |  |
|  | Elevation | -0.00218 | | 0.00439 | -0.50 | 0.632 |  |  |  |  |
|  | precipitation | 0.00747 | | 0.00285 | 2.62 | 0.031 | * |  |  |  |
| lm(formula = Species Richness ~ elevation + precipitation + temperature) | temperature | 0.96335 | | 1.26490 | 0.76 | 0.468 |  |  |  |  |
|  |  |  | |  |  |  |  |  |  |  |
|  | Residuals: |  | |  |  |  |  |  |  |  |
|  | Min 1Q Median 3Q Max |  | |  |  |  |  |  |  |  |
|  | -7.939 -4.470 -0.273 3.313 9.738 |  | |  |  |  |  |  |  |  |
|  |  |  | |  |  |  |  |  |  |  |
|  | Residual standard error: 6.75 on 8 degrees of freedom | | | |  |  |  |  |  |  |
|  | Multiple R-squared: 0.534 | Adjusted R-squared: 0.359 | | | |  |  |  |  |  |
|  | F-statistic: 3.05 on 3 and 8 DF, p-value: 0.0918 | | |  |  |  |  |  |  |  |
| lm(formula = Phylogenetic Diversity ~ elevation + precipitation + temperature) |  | | *Estimate* | *Std. Error* | *t value* | *p value* |  |  |  |  |
|  | Intercept | | 2.00714 | 2.88765 | 0.695 | 0.507 |  |  |  |  |
|  | Elevation | | -0.00040 | 0.00038 | -1.052 | 0.324 |  |  |  |  |
|  | precipitation | | 0.00058 | 0.00025 | 2.365 | 0.046 | * |  |  |  |
|  | temperature | | 0.01677 | 0.11027 | 0.149 | 0.885 |  |  |  |  |
|  |  | |  |  |  |  |  |  |  |  |
|  | Residuals: | | | | | | |  |  |  |
|  | Min 1Q Median 3Q Max | | | | | | |  |  |  |
|  | -0.870 -0.357 0.0.84 0.264 0.997 | | | | | | |  |  |  |
|  |  | | | | | | |  |  |  |
|  | Residual standard error: 0.588 on 8 degrees of freedom | | | | | | |  |  |  |
|  | Multiple R-squared: 0.559 Adjusted R-squared: 0.3929 | | | | | | |  | | |
|  | F-statistic: 3.37 on 3 and 8 DF, p-value: 0.07509 | | | | | | |  | | |
| Signif. codes: 0 ‘***’ 0.001 ‘**’ 0.01 ‘*’ 0.05 ‘.’ 0.1 ‘ ’ 1 |  |  | |  |  |  |  |  |  |  |

**Table S5.** Paired post-hoc comparisons between biogeographic units from the PERMANOVA analysis. Perms = Number of permutations. Significant value is shown in bold.

| **Groups** | **t** | **P_perm_** | **Perms** | **P_MC_** |
| --- | --- | --- | --- | --- |
| Equatorial Pacific, Equatorial Chocó | 17.419 | 0.096 | 10 | 0.066 |
| Equatorial Pacific, Western Andes | 20.214 | 0.105 | 10 | **0.029** |
| Equatorial Pacific, Equatorial Tumbes | 17.254 | 0.101 | 10 | 0.067 |
| Equatorial Chocó, Western Andes | 17.823 | 0.096 | 10 | 0.055 |
| Equatorial Chocó, Equatorial Tumbes | 1.764 | 0.103 | 10 | 0.061 |
| Western Andes, Equatorial Tumbes | 17.942 | 0.098 | 10 | 0.056 |
